# The Outcomes of Chest Wall Stabilization in Flail Chest Patients in the Intensive Care Unit

**DOI:** 10.14744/SEMB.2021.90947

**Published:** 2021-12-29

**Authors:** Servet Ozdemir, Selcuk Kose, Deniz Ozel Bilgi, Necati Citak, Mustafa Ozer Ulukan, Gulsum Oya Hergunsel

**Affiliations:** 1. Depatment of Thoracic Surgery, University of Health Sciences Turkey, Bakırköy Dr. Sadi Konuk Training and Research Hospital, Istanbul, Turkey; 2. Depatment of Anaesthesiology and Reanimation, University of Health Sciences Turkey, Bakırköy Dr. Sadi Konuk Training and Research Hospital, Istanbul, Turkey; 3. Department of Cardiovascular Surgery, Istanbul Medipol University Faculty of Medicine, Istanbul, Turkey

**Keywords:** Flail chest, invasive mechanical ventilation, mortality, multi-trauma, rib fixation

## Abstract

**Objectives:**

In this study, the effect of multi-trauma on treatment results in flail chest patients who underwent chest wall stabilization was investigated.

**Methods:**

The data of thirty-six flail chest cases between the ages of 18–79 who were consulted for thoracic surgery were retrospectively analyzed in the study. The presence of flail chest in the patients was confirmed by thoracic surgeons, and the multi-traumas were confirmed through the diagnoses made by specialist physicians reexamining clinical methods.

**Results:**

It was found that 27 (75%) of flail chest cases evaluated had multi-trauma, and 3 (8.3%) of the cases had mortality in the study. It was found that the duration of the intensive care unit stay and the number of days on invasive mechanical ventilation of the cases were positively correlated with the number of surgical areas exposed to trauma (p<0.05). According to the univariate binary logistic regression analysis, it was found that the total number of rib fractures (OR = 1.44, p=0.055), the number of fixed ribs (OR = 0.76, p=0.558), the number of plates placed for fixation (OR = 0.70, p=0.368), and the number of additional trauma areas outside the thorax (OR = 6.76, p=0.076) were not statistically significant in increasing the mortality risk.

**Conclusion:**

Considering that multi-trauma is an effective factor in the prolongation of the duration of treatment, the management of traumas with different specialties can positively affect the treatment results and reduce the risk of mortality.

Flail chest is a serious clinical entity that occurs as a result of the fracture of at least 3 ribs in 2 places in the thorax due to trauma and causes paradoxical breathing by forming an unstable chest wall in patients.^[1]^ In a published study investigated national trauma data in the USA, the incidence of the flail chest was found 1.4% in more than one million chest trauma patients.^[2]^ Moreover, 4.5% of patients with the flail chest were treated with surgical operation, and the rate of surgical operation has increased.^[3]^ The mortality rate was 1.5% in flail chest cases who underwent rib stabilizations, whereas the mortality rate was 12.4% in the flail chest cases treated without surgery.^[4]^ Surgery in flail chest cases is still controversial, although some researchers stated that surgical interventions result in a lower rate of pneumonia, fewer days on mechanical ventilation, and a shorter hospital stay.^[5-7]^

It has been stated that the presence of multi-trauma in flail chest cases affects the results of the treatment that were intensive care unit (ICU) stay length, duration of hospitalization, and ventilation. However, the effect of multi-trauma on the results of the methods used for the treatment of flail chest has not been adequately studied.^[8]^ Therefore, we aimed to investigate whether the presence of multi-trauma on treatment results in flail chest patients who had chest wall stabilization in the past was examined retrospectively.

## Methods

**Figure 1.**
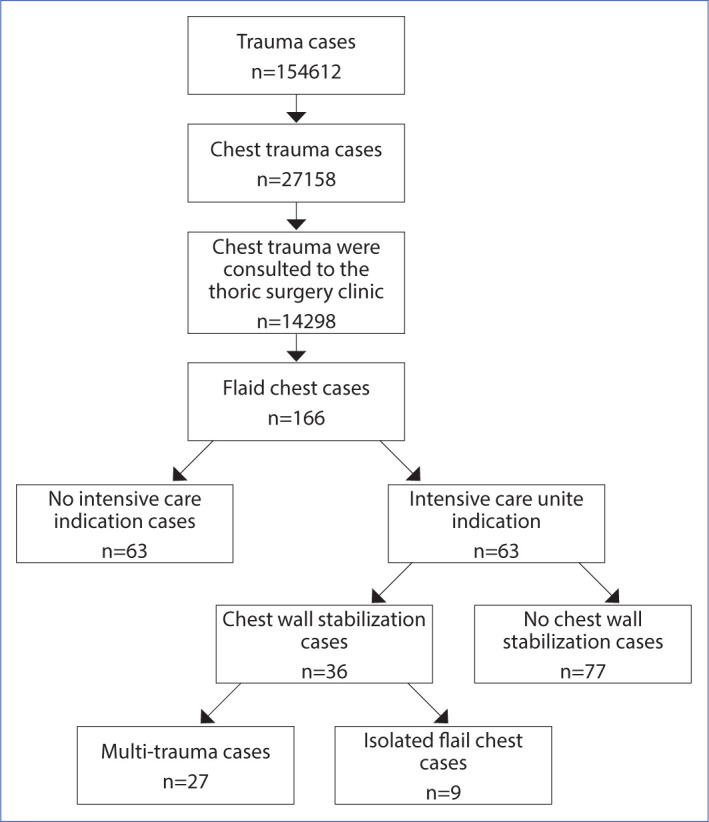
Flow the present study.

In this study, the medical records in a training and research hospital that provide a dense population in Turkey, and it was detected that 27.158 (17.6%) cases had chest trauma among 154.612 trauma cases between January 1^st^, 2015 and December 31^st^, 2019 concerning the examination. There were 14.298 (52.6%) cases with chest trauma, and 166 of these cases were diagnosed as a flail chest. Flail chest was evaluated as patients with at least 3 rib fractures in 2 or more places rib fractures. Furthermore, paradoxical breathing was present in those patients. In sixty-three of the cases with a diagnosis of flail chest, there was no indication of admit the ICU. The remaining 103 cases were admitted to the ICU. Thirty-six of those patients who were admitted to the ICU underwent chest wall stabilization were investigated retrospectively. They had respiratory instability despite mechanical ventilation support, therefore, were performed chest wall stabilization (Fig. 1). Twenty-seven patients who underwent chest wall stabilization had a multi-trauma, whereas the remaining nine patients had an isolated thoracic trauma.

The data of patients who were diagnosed with flail chest but whose operation was not approved by the patient or legal representative were not included in the study. Before the study, approval was obtained from the ethics committee of the hospital for retrospective examination of 36 cases for scientific purposes, with the decision of the ethics committee numbered; 2020/344-İstanbul Bakırköy Dr. Sadi Konuk Training and Research Hospital Clinical Research Ethics Committee.

### Assessment and Treatment

**Figure 2.**
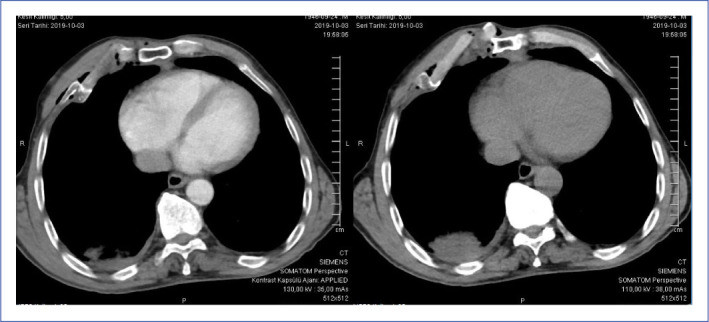
Thoracic CT of a flail chest case with rib fracture.

When the data were analyzed retrospectively, it was seen that the diagnosis of the cases evaluated in the study was recorded in the thoracic surgery clinic with the code ICD-10: S22.5. Therefore, the diagnoses of the cases evaluated in this study were determined according to the rule of the fraction that is, according to ICD-10: S22.5, at least 3 ribs in 2 places in the chest. The medical records of the patients were reviewed and multi-traumas determined by other specialties were re-recorded for the study. Thorax CT results of the patients were checked again by examining the patient files. In addition, the results of the medical methods applied to determine the additional trauma areas outside the thorax (cranial, vertebral, abdominal, and extremity) that may occur in the cases, the clinical and radiological results of the traumatic area were examined for research and the data were recorded. With this method, all routine physical examinations and medical examinations (routine blood tests, blood gas levels, saturation results, X-ray radiography ultrasound, computed tomography (Fig. 2), and MRI) performed on a patient with multi-trauma were reviewed.

Based on the examination of the patients’ records, it was found out that the medical follow-up and treatment of the patients who were taken to the ICU after the operation was performed in the thoracic surgery service. Besides that, it was evaluated that chest physiotherapy was carried out to patients who continued their medical treatment in the thoracic surgery clinic.

**Figure 3.**
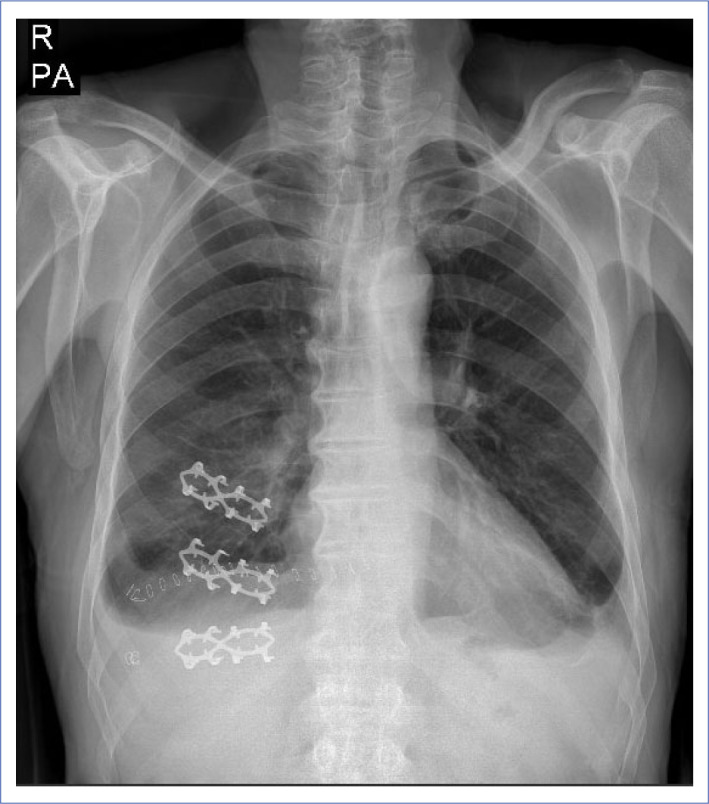
Posteroanterior chest radiograph of a post-operative case.

Since nine of the cases evaluated in the study had isolated thoracic trauma, it was stated that these cases were primarily performed using rib fixation material which is specifically named 6-segment shape memory alloys (IAWA®, Chine), and the patients were followed by thoracic surgeons from the ICU to the time of discharge (Fig. 3). It was observed that rib fixation was performed in 27 patients with multi-trauma in coordination with relevant specialists and the patients were followed by other relevant clinical branches. In an attempt to record the data of the patients, a questionnaire consisting of demographic (age, gender, side of the trauma, the presence of intubation, the day of stay in ICU, the days of intubation after surgery, the presence of surgical interventions outside the thorax, mortality, and/or discharge) and clinical characteristics (cause of the trauma, additional trauma area, duration of treatment, etc.) was used by the researchers.

### Surgical Procedure

All of the patients were those who were provided with lung ventilation by inserting a single-lumen endotracheal tube with general anesthesia. In the cases, a vertical (between 4^th^ and 8^th^ ribs) extended incision was made on the anterior, middle, or axillary line between the M. Pectoralis major and M. Latissimus dorsi muscles in the lateral decubitus position according to the location of the fracture lines. In addition, the M. Serratus muscle was separated by the muscle-sparing technique in the vertical plane according to the size of the incision and the number of rib fracture segments. As a result of these procedures, it was observed that ribs and fracture lines could be reached. After the detection of the fracture lines by visual examination and palpation, it was observed that the periosteum layer over the rib fracture was separated using a periosteum scraper hand tool. When the patient files were examined, it was found out that the fracture line was manually put together and fixed with a costal plate.

**Figure 4.**
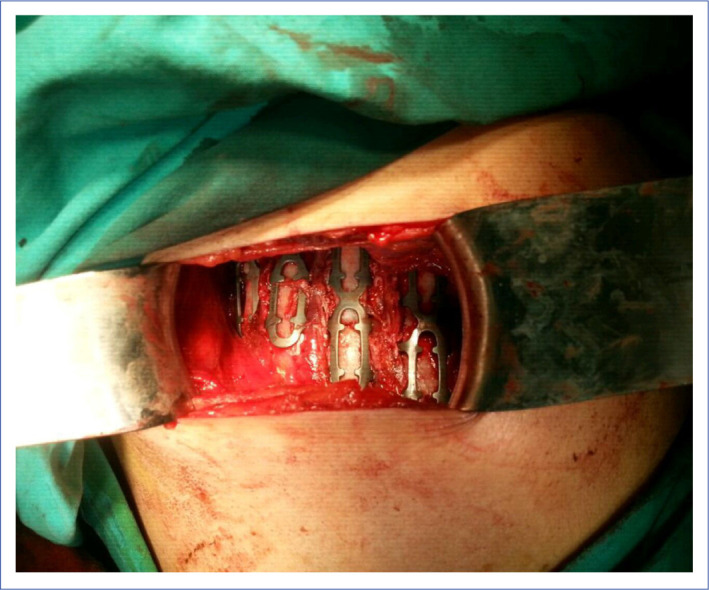
Intraoperative rib fractures stabilization of a flail chest case.

In patients with intra-thoracic pathology accompanying rib fractures, it was also evaluated that the thorax was accessed by opening the intercostal muscle and both lung parenchyma repair and hematoma drainage procedures were performed (Fig. 4).

### Statistical Analysis

The demographic and clinical characteristics of the flail chest cases were evaluated by descriptive statistical analyzes such as number, mean, and percentage. Numerical data between flail chest cases with and without multi-trauma were analyzed by the Mann Whitney U test, and proportional data were analyzed with Chi-square Analysis. The relationship between the number of surgical areas exposed to trauma, the duration of ICU stay, and the number of days on invasive mechanical ventilation was analyzed using Spearman Correlation Analysis. Univariate Binary Logistic Regression analysis was used to evaluate whether demographic and clinical features increase the risk of multi-trauma and mortality. The properties of variables affecting the risk of multi-trauma and mortality were analyzed using Odds Ratio (OR) and 95% Confidence Interval (CI). The level of significance for all analyzes was set as p<0.05 and IBM SPSS 22.0 program was used in the analysis.

## Results

The mean age of the cases evaluated in the study was 44.6 (minimum = 18, maximum = 79). Multi-trauma was found in 27 (75%) of the flail chest cases in the study. Sixteen of the cases (44.4%) had one additional trauma area, 10 (27.8%) of them had two additional trauma areas, and one (2.8%) had three additional trauma areas. Moreover, intraoperative 8 (22.2%) cases had lung laceration, and 19 (52.8%) cases had an intra-thoracic hematoma (Table 1).

**Table 1.** Comparison of demographic characteristics and cause of trauma between cases with and without multi-trauma

	**No**		**Yes**		**P**
	**Mean/n**	**Median/%**	**Mean/n**	**Median/%**	
Age	43.56	39.00	44.96	44.00	0.886^a^
Gender					
Male	6	66.7	19	70.4	0.835^b^
Female	3	33.3	8	29.6	
Cause of trauma					
Pedestrian traffic accident	2	22.2	12	44.4	0.149^c^
In-vehicle traffic accident	3	33.3	8	29.6	
Gunshot	1	11.1	0	0.0	
CPR	1	11.1	0	0.0	
Pounding	1	11.1	1	3.7	
Fall	1	11.1	6	22.2	

^a^Mann Whitney U test, ^b^Pearson Chi-square analysis, ^c^Fisher’s Exact Chi-square analysis.

In the comparison between the flail chest cases with and without multi-trauma, it was found that the rates of the variables were not statistically significantly different, except for the rates of chest drainage tube insertion (p=0.010) and the chest trauma side (p=0.04) (Table 2).

**Table 2.** Comparison of clinical and treatment characteristics between flail chest cases with and without multi-trauma.

	**No**		**Yes**		**P**
	**Mean/n**	**Median/%**	**Mean/n**	**Median/%**	
Duration of stay in intensive care unit	7.22	5.00	18.81	6.00	0.233a
Intubation					
No	2	22.2	4	14.8	0.606b
Yes	7	77.8	23	85.2	
Number of days on mechanical ventilation	3.89	1.00	12.89	3.00	0.263a
Number of days on mechanical ventilation after fixation	1.33	1.00	10.59	2.00	0.146a
Tracheotomy					
No	9	100.0	19	70.4	0.160c
Yes	0	0.0	8	29.6	
Number of rib fractures fixed	4.56	5.00	4.19	4.00	0.312a
Mortality					
No	9	100.0	24	88.9	0.558c
Yes	0	0.0	3	11.1	
Sternum fracture					
No	9	100.0	23	85.2	0.553c
Yes	0	0.0	4	14.8	
Subcutaneous emphysema					
No	7	77.8	15	55.6	0.236b
Yes	2	22.2	12	44.4	
Pulmonary contusion					
No	4	44.4	14	51.9	0.700b
Yes	5	55.6	13	48.1	
Right-sided rib fracture	1.78	.00	4.37	5.00	0.056a
Left-sided rib fracture	4.78	5.00	4.85	5.00	0.971a
Total rib fracture	6.56	7.00	9.22	9.00	0.067a
Hemopneumothorax					
No	2	22.2	1	3.7	0.148c
Yes	7	77.8	26	96.3	
Chest drainage tube insertion					
No	4	44.4	2	7.4	0.010b
Yes	5	55.6	25	92.6	
Thoracotomy					
No	5	55.6	15	55.6	0.999b
Yes	4	44.4	12	44.4	
Pleural catheter was inserted					
No	9	100.0	26	96.3	0.999c
Yes	0	0.0	1	3.7	
Number of plate	5.11	5.00	4.59	4.00	0.233a
Number of days until the operation	4.67	2.00	5.78	4.00	0.590a
Number of days in service	8.89	7.00	7.15	7.00	0.279a
Additional surgical operation outside the thorax					
No	9	100.0	10	37.0	0.001c
Yes	0	0.0	17	63.0	
Abdomen					
No	9	100.0	18	66.7	0.076c
Yes	0	0.0	9	33.3	
Orthopedics					
No	9	100.0	19	70.4	0.168c
Yes	0	0.0	8	29.6	
Neurosurgery					
No	9	100.0	22	81.5	0.302c
Yes	0	0.0	5	18.5	
Chest trauma side					
Right	2	22.2	5	18.5	0.046c
Left	6	66.7	7	25.9	
Bilateral	1	11.1	15	55.6	
Video-assisted thoracoscopic surgery					
No	9	100.0	26	96.3	0.099c
Yes	0	0.0	1	3.7	

It was found that age (OR = 1.01; 95% CI: 0.96–1.06, p = 0.818), male gender (OR = 2.67; 95% CI: 0.24–5.96, p = 0.835), fall (OR = 2.87; 95% CI: 0.24–22.09, p = 0.475), pedestrian traffic accident (OR = 0.84; 95% CI: 0.17–4.23, p = 0.835), and in-vehicle traffic accident (OR = 2.80; 95% CI: 0.49–16.04, p = 0.218) did not significantly affect the risk of multitrauma (Table 3).

**Table 3.** Univariable logistic regression analysis linked to multi-trauma risk

	**OR**	**95% CI**	**p**
Age	1.01	0.96–1.06	0.818
Gender (Male)	2.67	0.24–5.96	0.835
Fall	2.87	0.24–22.09	0.475
In-vehicle traffic accident	0.84	0.17–4.23	0.835
Pedestrian traffic accident	2.80	0.49–16.04	0.248

OR: Odds ratio; CI: Confidence interval.

A statistically significant positive correlation was found between the total number of surgical areas exposed to trauma, the duration of stay in the ICU (r = 0.368, p = 0.027), and the number of days on invasive mechanical ventilation (r = 0.450, p = 0.006). It was detected that there was a statistically significant positive correlation between the total number of rib fractures of the cases, the duration of stay in the ICU (r = 0.448, p = 0.006), and the number of days on invasive mechanical ventilation (r = 0.345, pP = 0.040).

The mortality rate was 8.3% (n = 3). They were male, had a multi-trauma, and invasively mechanically ventilated. Pulmonary contusion, hemopneumothorax, head trauma, and spine trauma were also detected in all cases with mortality. The results of the variables that can be effective in increasing the mortality risk are shown in detail in Table 4.

**Table 4.** Univariable logistic regression analysis linked to mortality

	**OR**	**95% CI**	**p**
Age	1.01	0.93–1.08	0.904
In-vehicle traffic accident	1.15	0.09–14.19	0.913
Pedestrian traffic accident	3.50	0.27–42.77	0.327
Duration of stay in intensive care unit	1.01	0.97–1.05	0.811
Number of days on invasive mechanical ventilation	1.02	0.97–1.06	0.456
Number of days on invasive mechanical ventilation after fixation	1.02	0.98–1.06	0.396
Tracheotomy (yes)	1.86	15–23.58	0.633
Number of stabilized rib fractures	0.76	0.35–1.77	0.558
Sternum fracture	31.00	1.90–506.77	0.016
Subcutaneous emphysema	3.50	0.27–42.77	0.327
Total number of rib fractures	1.44	0.99–2.09	0.055
Thoracotomy	2.71	0.22–32.99	0.433
Number of plates	0.70	0.32–1.52	0.368
Number of days until the operation	0.79	0.48–1.30	0.362
Additional surgical operation outside the thorax	0.53	0.04–6.44	0.619
Number of traumas	6.76	0.82–55.93	0.076

OR: Odds ratio; CI: Confidence interval.

## Discussion

It was found that 75% of patients, who were followed up in ICU and provided mechanical ventilation support, in flail chest cases had multi-trauma, and all cases with mortality (8.3%) were male and had multi-trauma, oral tracheal intubation, pulmonary contusion, hemopneumothorax, head trauma, and spine trauma in the present study. It was detected that the demographic and clinical characteristics of flail chest cases with and without multi-trauma were not different. However, it was found that the number of additional surgical areas exposed to trauma outside the thorax in patients with multi-trauma positively correlated with the duration of the ICU stay and the number of days on invasive mechanical ventilation. According to the Univariate Binary Logistic Regression analysis, it was found that the presence of sternum fracture, subcutaneous emphysema, the presence of thoracotomy, the total number of rib fractures, and the number of additional surgical trauma areas outside the thorax may increase the risk relatively, but this increase was not statistically significant.

Marasco et al. (2016) evaluated 46 flail chest patients and found that 31 (67.4%) of the cases had multi-trauma, and these traumas included spine, and head trauma. In another study, approximately, half of the flail chest cases were found to have additional trauma.^[2]^ Considering that in flail chest cases evaluated in the present study, it can be said that the rate of multi-trauma is 75% and that nearly half of these cases suffered head and spine trauma, multi-trauma are, therefore, prevalent in flail chest patients and the findings obtained from the study are compatible with the literature. Researchers stated that multi-trauma seen in flail chest cases had negative effects on the treatment process; however, the effect of multi-trauma on treatment results was not sufficiently investigated.^[7,8]^ According to the results obtained from the current study, it can be said that additional medical and surgical treatments are more common in flail chest patients with multi-trauma, and the length of the treatment period of these patients is relatively longer, but this difference is not statistically significant. Therefore, conducting studies with larger samples to better understand the effect of the presence of multi-trauma on treatment results in flail chest patients may contribute to the literature.

In this study, it was found that the mortality rate was 8.3% in flail chest patients who underwent chest wall stabilization, all cases with mortality were performed oral-tracheal intubation, and they had multi-trauma, pulmonary contusion, and hemopneumothorax. The mortality rate in flail chest cases without multi-trauma was 5.6% whereas thoracotomy, mechanical ventilation, cardiac contusion, and sternal fracture have been found to increase the risk of mortality in isolated flail chest cases.^[2]^ In a systematic review, it was reported that the mortality rate in flail chest cases ranged from 16% to 25%.^[9]^ Therefore, according to the above studies, it can be said that the mortality rate in flail chest patients with multi-trauma is higher than in isolated flail chest patients and lower than the studies examining cases with multi-trauma. In this study, patients admitted to a single center were evaluated. In addition, the number of cases is limited. Therefore, good management of additional traumas of the flail chest cases with multi-trauma may reduce the mortality rate relatively in this patient group.

In the study, age, the total number of rib fractures, the number of fixed ribs, the number of plates placed, and the number of additional surgical trauma areas outside the thorax increased the risk of mortality relatively, but this increase was not statistically significant. In some studies, it has been reported that the severity of the trauma, age characteristics, hemopneumothorax, and receiving invasive mechanical support affect the duration of hospital stay in flail chest patients, but do not affect mortality.^[10,11]^ In a study examining unilateral flail chest cases, it was found that age characteristics and severity of trauma increased the risk of mortality, but additional thoracic traumas and multi-trauma did not have a significant effect on mortality risk.^[12]^ Some studies have found that the primary predictors of mortality are multi-trauma, bilateral flail chest injuries, hemopneumothorax, and advanced age.^[7,13,14]^ The results of the studies in the literature are that additional trauma in flail chest cases with multi-trauma does not significantly increase the mortality risk. However, in many different studies, the fact that trauma severity is a predictor of mortality has shown that the duration of treatment of flail chest cases with multiple traumas is longer, and the treatment applications are more diverse. Similarly, in the present study, the correlation between the number of traumas and duration of treatment, and the number of days on mechanical ventilation may support this view. However, it can be kept in mind that clinical features and treatment properties are relatively different between cases with and without multi-trauma, and this difference is not statistically significant.

The limited number of participants in the study and the limited number of isolated flail chest cases should be considered among the limitations of the study.

## Conclusion

It was deduced that the presence of multi-trauma in flail chest cases was relatively effective in increasing the mortality risk. The more surgical areas exposed to trauma in flail chest cases, the longer the duration of stay in ICU and the more days on mechanical ventilation. Clinical features and treatment methods of flail chest patients with or without multi-trauma are similar. However, it should be kept in mind that the presence of multi-trauma, the presence of hemopneumothorax, and the support of the patient with invasive mechanical ventilation require a multidisciplinary approach to decrease the mortality rate in flail chest patients. Moreover, it can be said that the flail chest can be fixed surgically with a multidisciplinary approach in patients with multi-trauma, and surgical treatment is an important option for these patients. As the number of rib fractures increases, the duration of stay in the MV and ICU increases, so patients with multiple rib fractures should be given special care.

### Disclosures

**Ethics Committee Approval:** Istanbul Bakirkoy Dr. Sadi Konuk Training and Research Hospital Clinical Research Ethics Committee. No: 2020/344.

**Peer-review:** Externally peer-reviewed.

**Conflict of Interest:** None declared.

**Funding:** This research did not receive any specific grant from funding agencies in the public, commercial, or not-for-profit sectors.

**Authorship Contributions:** Concept – S.O., S.K., N.C., O.G.H.; Design – S.O.; Supervision – S.O., S.K., O.G.H.; Materials – S.O., D.O.; Data collection &/or processing – S.O., S.K., D.O.; Analysis and/or interpretation – S.O., N.C.; Literature search – S.O., N.C.; Writing – S.O., M.O.U.; Critical review – S.O., M.O.U.
